# Optical coherence tomography angiography and Humphrey field analyser for macular capillary non-perfusion evaluation in branch retinal vein occlusion

**DOI:** 10.1038/s41598-021-84240-7

**Published:** 2021-02-25

**Authors:** Hiroko Terashima, Fumiki Okamoto, Hiruma Hasebe, Eriko Ueda, Hiromitsu Yoshida, Takeo Fukuchi

**Affiliations:** 1 Division of Ophthalmology and Visual Science, Graduate School of Medical and Dental Sciences, Niigata University, 1-757 Asahimachi-dori, Chuo-ku, Niigata City, Niigata 951-8510 Japan; 2 Department of Ophthalmology, Faculty of Medicine, University of Tsukuba, 1-1-1 Tennoudai, Tsukuba, Ibaraki 305-8575 Japan

**Keywords:** Medical research, Diseases, Eye diseases, Retinal diseases

## Abstract

We non-invasively evaluated macular non-perfused areas (m-NPAs) of branch retinal vein occlusion (BRVO) using optical coherence tomography (OCT) angiography and the Humphrey visual field analyser 10-2 programme (HFA 10-2). We enrolled 30 patients (30 eyes) with macular oedema secondary to BRVO. OCT angiography was used to photograph the macula at 6 × 6-mm; sizes of m-NPAs in the superficial capillary plexus (SCP) and deep capillary plexus (DCP) were measured in four areas. For HFA 10-2, we divided the actual measurement threshold of 68 points into four areas and calculated the mean central visual field sensitivity (CVFS). The correlation between the mean m-NPA and mean CVFS (dB) in each area was examined. There was a strong correlation between the m-NPA of each region detected in SCP and DCP, and the mean CVFS of each corresponding area (SCP: r = − 0.83, r = − 0.64, r = − 0.73, and r = − 0.79; DCP: r = − 0.82, r = − 0.71, r = − 0.71, and r = − 0.70), p values were < 0.001 for all. m-NPAs were associated with decreased visual field sensitivity in BRVO. Non-invasive m-NPA evaluation was possible using OCT angiography and HFA 10-2.

## Introduction

Branch retinal vein occlusion (BRVO) is the second most commonly occurring retinal vascular disease after diabetic retinopathy (DR). It is associated with complications related to cystoid macular oedema (CME), serous retinal detachment (SRD), and macular non-perfused areas (m-NPAs)^1,2^, and leads to the deterioration of visual acuity and permanent visual field disturbance. Fluorescence angiography (FA) is used for the evaluation of NPA absence or presence. However, since it is an invasive examination with the possibility of anaphylactic shock (due to dye injection)^3^ its usage rates have somewhat reduced; therefore, there is a need for a non-invasive evaluation method for NPAs. Several non-invasive methods have been reported for the evaluation of the non-perfusion status of the retina without angiography^4,5^. Yong et al. reported that confocal red-free imaging is a safe and non-invasive method for the effective plotting of retinal non-perfusion for DR and retinal vein occlusion^6^.

In recent years, optical coherence tomography (OCT) angiography has made detailed observation of the retinal vasculature possible^7–10^. Some studies have analysed if the findings obtained on OCT angiography are similar to those observed on FA^11–14^.

Meanwhile, the evaluation of the visual field using the Humphrey visual field analyser 10-2 programme (HFA 10-2) has attracted attention in glaucoma settings^15–18^. In BRVO, the inner layer of the retina becomes thin due to retinal ischaemia, and the sizes of the ganglion cell complex, ganglion cell-inner plexiform layer, and retinal nerve fibre layer decrease^5,19,20^. Since the thickness of the ganglion cell complex is associated with visual field sensitivity^21^, it is expected that HFA 10-2, which can accurately measure the central visual field sensitivity (CVFS) of the macula, can evaluate the degree of macular ischaemia. HFA is an examination device that has been installed in every facility in our country, yet it is not widely used in routine practice for retinal diseases. Focusing on the versatility of HFA, we aimed to introduce HFA10-2 as a suitable and accessible tool to detect macular ischaemia in BRVO in clinical practice. To the best of our knowledge, no study to date has focused on the evaluation of the relationship between m-NPA and CVFS of the macula in BRVO using HFA. Therefore, we retrospectively investigated whether m-NPAs can be assessed non-invasively through comparisons of the m-NPA values obtained using OCT angiography and HFA 10-2 for resolved BRVO-CME.

## Results

The study sample comprised 30 eyes in 30 participants (mean age 67.7 ± 9.2 years, range 44–81 years). Table 1 shows the clinical characteristics of the patients with BRVO. The mean best-corrected visual acuity (BCVA) at the initial visit was 0.45 ± 0.29 (Snellen equivalent: 20/57), and that at evaluation was 0.05 ± 0.28 (Snellen equivalent: 20/22). The mean central retinal thickness (CRT) at the initial visit was 537 ± 170 μm, and that at evaluation was 257 ± 38 μm.

**Table 1:** Clinical characteristics of patients with macular oedema secondary to branch retinal vein occlusion.

	BRVO (n = 30)
Age (years)	67.7 ± 9.2
Sex (male/female), no (%)	10 (33.3)/20 (66.7)
BCVA at initial visit (logMAR) (Snellen equivalent)	0.45 ± 0.24 (20/57)
BCVA at evaluation (logMAR) (Snellen equivalent)	0.05 ± 0.28 (20/22)
CRT at initial visit (μm)	537 ± 170
CRT at evaluation (μm)	257 ± 38
Evaluation time (months)	10.0 ± 6.2
Location of vein occlusion, no (%) (superior/inferior)	16 (53.3)/14 (46.7)
Occlusion type, no (%) (major/macular)	24 (80.0)/6 (20.0)
Intravitreal injection (IVR/IVA), no (%)	25 (83.3)/5 (16.7)

Values are presented as means ± standard deviation.

*BRVO* branch retinal vein occlusion, *BCVA* best-corrected visual acuity, *logMAR* logarithm of the minimum angle of resolution, *CRT* central retinal thickness, *IVR* intravitreal ranibizumab, *IVA* intravitreal aflibercept injection.

### Macular non-perfusion area in OCT angiography

An m-NPA > 2 disc diameters in the superficial capillary plexus (SCP) and deep capillary plexus (DCP) was detected in 16 eyes (53%) and 20 eyes (67%), respectively. The mean area of the SCP-NPA was 5.1 ± 4.6 mm^2^ and that of the DCP-NPA was 6.3 ± 5.0 mm^2^. On comparing the m-NPA values of the SCP and DCP, the size of m-NPA in DCP was found to be slightly more extensive than that of SCP, but no significant difference was recognised (p = 0.34). Table 2 shows the comparison of the mean m-NPA values in each area of the SCP and DCP on OCT angiography; no significant differences were found.

**Table 2:** Comparison of the mean m-NPA in each area of the SCP and DCP on OCT angiography.

m-NPA (mm^2^)	Superior temporal	Superior nasal	Inferior temporal	Inferior nasal
SCP	2.01 ± 2.82	0.89 ± 1.95	1.47 ± 2.70	0.68 ± 1.41
DCP	2.17 ± 2.88	1.34 ± 2.23	1.70 ± 2.90	1.04 ± 2.00
P*	0.739	0.559	0.675	0.475

Values are presented as means ± standard deviation.

*m-NPA* macular non-perfused area, *OCT* optical coherence tomography, *SCP* superficial capillary plexus, *DCP* deep capillary plexus.

*P value by unpaired t test.

### Central visual field sensitivity in HFA 10-2

Table 3 shows the comparison of the mean CVFS values between the BRVO-affected macular area and non-affected macular area for the temporal and nasal lesion. The mean temporal CVFS was 26.8 ± 4.0 dB in the affected macular area and 30.1 ± 1.7 dB in the non-affected macular area. Similarly, the mean nasal CVFS was 23.1 ± 7.4 dB in the affected macular area and 30.0 ± 1.7 dB in the non-affected macular area. The CVFS of the BRVO-affected area was significantly lower on both, the temporal lesion and nasal lesion than that of the non-affected area (p < 0.001, p < 0.001, respectively).

**Table 3:** Comparison of the mean central visual field sensitivity of the BRVO-affected macular area and the non-affected macular area.

	Affected macular area	Non-affected macular area	P*
Temporal CVFS (dB)	26.8 ± 4.0	30.1 ± 1.7	< .001
Nasal CVFS (dB)	23.1 ± 7.4	30.0 ± 1.7	< .001

Values are presented as means ± standard deviation.

*CVFS* central visual field sensitivity, *BRVO* branch retinal vein occlusion.

*P value by unpaired t test.

### Correlation between m-NPA on OCT angiography and CVFS

**Figure 1:**
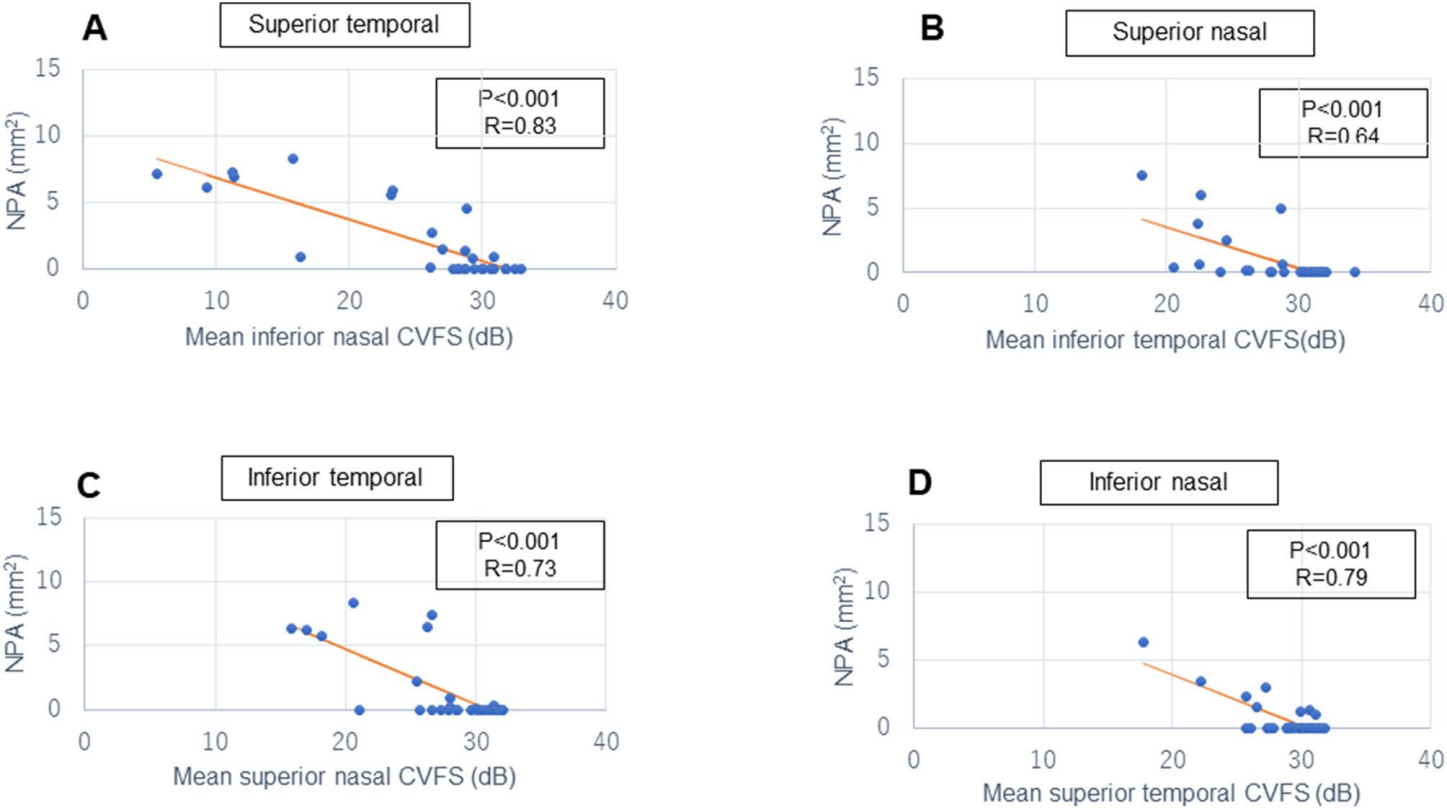
Scatter plots of the correlation between macular m-NPAs on OCT angiography in the SCP and corresponding CVFS values on HFA 10-2. (**A**) m-NPA in the superior temporal region and mean inferior nasal CVFS (**B**) m-NPA in the superior nasal region and mean inferior temporal CVFS (**C**) m-NPA in the inferior temporal region and mean superior nasal CVFS (**D**) m-NPA in the inferior nasal region and mean superior temporal CVFS. *m-NPA* macular non-perfused areas, *OCT* optical coherence tomography, *SCP* superficial capillary plexus, *HFA 10-2* Humphrey visual field analyser 10-2 programme, *CVFS* central visual field sensitivity.

**Figure 2:**
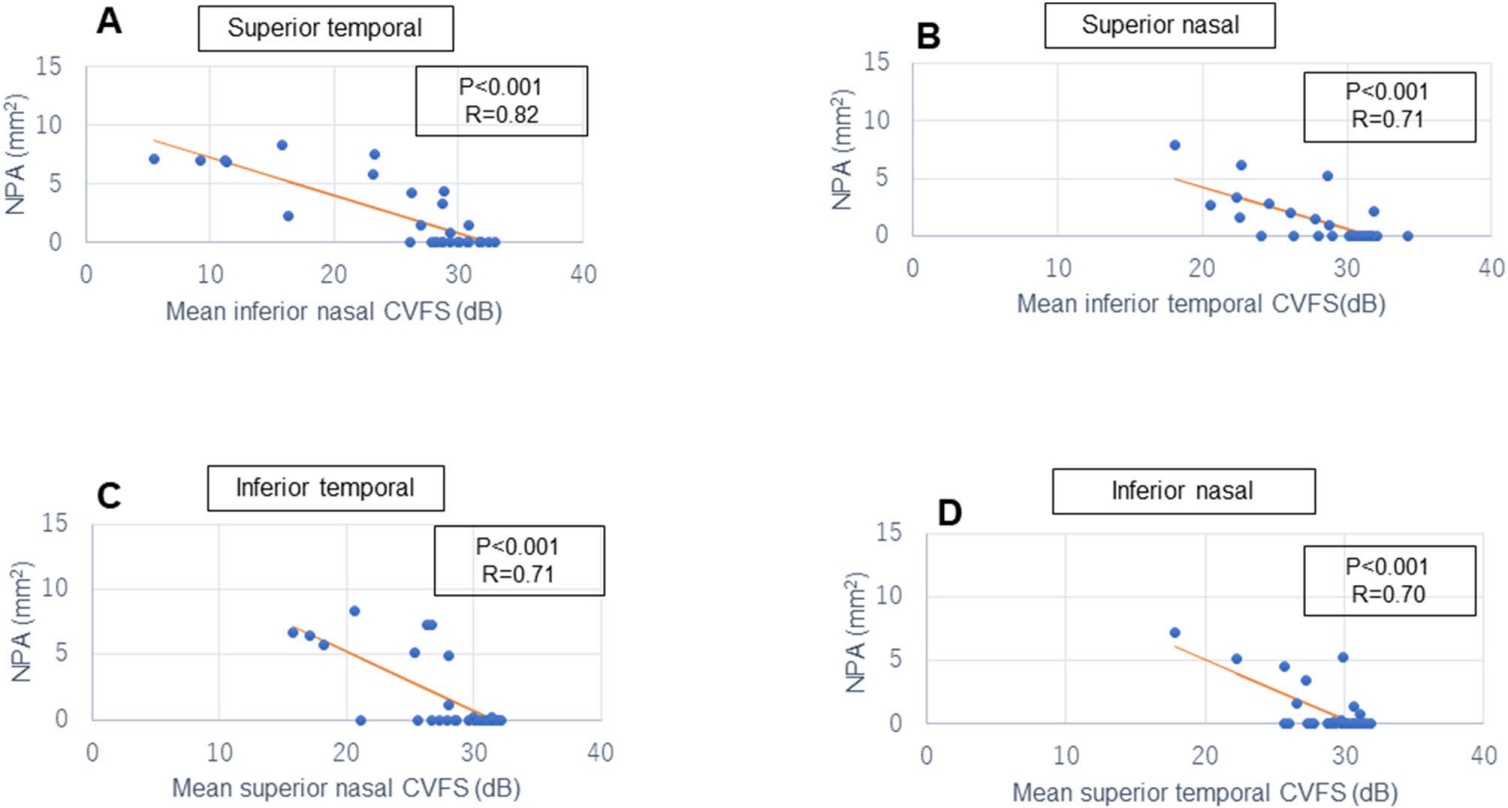
Scatter plots of the correlation between macular m-NPA on OCT angiography in the DCP and corresponding CVFS values on HFA 10-2. (**A**) m-NPA in the superior temporal region and mean inferior nasal CVFS (**B**) m-NPA in the superior nasal region and mean inferior temporal CVFS (**C**) m-NPA in the inferior temporal region and mean superior nasal CVFS (**D**) m-NPA in the inferior nasal region and mean superior temporal CVFS. *m-NPA* macular non-perfused areas, *OCT* optical coherence tomography, *DCP* deep capillary plexus, *HFA10-2* Humphrey visual field analyser 10-2 programme, *CVFS* central visual field sensitivity.

Figures 1, 2 and Supp Table 1 show the correlation between the m-NPA on OCT angiography and CVFS on HFA 10-2. There was a strong correlation between the m-NPA detected in the SCP and the corresponding CVFS in each of the four areas (superior temporal: r = − 0.83, superior nasal: r = − 0.64, inferior temporal: r = − 0.73, inferior nasal: r = − 0.79 and all p < 0.001) (Fig. 1). Similarly, we found a high negative correlation in all four areas between the m-NPA of the DCP and the corresponding CVFS (superior temporal: r = − 0.82, superior nasal: r = − 0.71, inferior temporal: r = − 0.71, inferior nasal: r = − 0.70 and all p < 0.001) (Fig. 2).

### Reproducibility of HFA 10-2 in BRVO patients

The mean age of the 11 BRVO patients was 66.0 ± 10.7 years. The mean CVFS in the affected macular areas on tests 1 and 2 were 28.8 ± 2.1 dB and 28.8 ± 2.6 dB, respectively, and the mean CVFS in the non-affected macular area on tests 1 and 2 were 31.6 ± 1.9 dB and 31.2 ± 1.6 dB, respectively. The mean CVFS values of the affected and non-affected macular areas showed no significant difference between tests 1 and 2 data (p = 0.12 and p = 0.23, respectively, using the Wilcoxon signed rank test), thus confirming reproducibility.

## Discussion

In the present study, we found that OCT angiography was useful in the evaluation of m-NPAs in BRVO, and that its use in combination with HFA 10-2 allowed for the evaluation of the vascular perfusion state and macular function of the corresponding site. Macular ischaemia in BRVO affects vision-related prognoses. In addition, the extensive formation of non-perfused areas carries the risk of developing retinal neovascularisation. It can cause vitreous haemorrhage and retinal detachment. Therefore, although retinal ischaemia can be appropriately evaluated, since invasive examinations such as FA are now being avoided, a method for the non-invasive evaluation of retinal ischaemia is required. OCT angiography, which was first used in 2015, has made it possible to non-invasively assess the retinal blood vessels in the macular region by stratification^6–9^. Besides, as a method for the evaluation of ischaemia through morphological examinations, it has been shown that the area of the ischaemic retina decreases in terms of the thickness of the ganglion cell-inner plexiform layer and retinal nerve fibre layer^20^. In the OCT en-face image, the dark areas are highly correlated to the NPA size obtained on FA^4^. Also, regarding the evaluation of NPA using functional examinations, Chee et al. reported that areas of capillary non-perfusion in DR demonstrated by FA was closely associated with reduced retinal sensitivity detected by the HFA 30-2 threshold test^22^. In the HFA30-2 programme, the central 10 degrees are measured at 6-degree intervals; therefore, only 12 points are measured. The 10-2 programme, on the other hand, measures at two-degree intervals, allowing for visual field evaluation specialised for the CVFS. Evaluation of macular ischaemia using microperimetry was recently performed^13,19,23–26^. However, at present, there is a lack of detailed information on the use of HFA 10-2 with specialisation in the macula.

**Figure 3:**
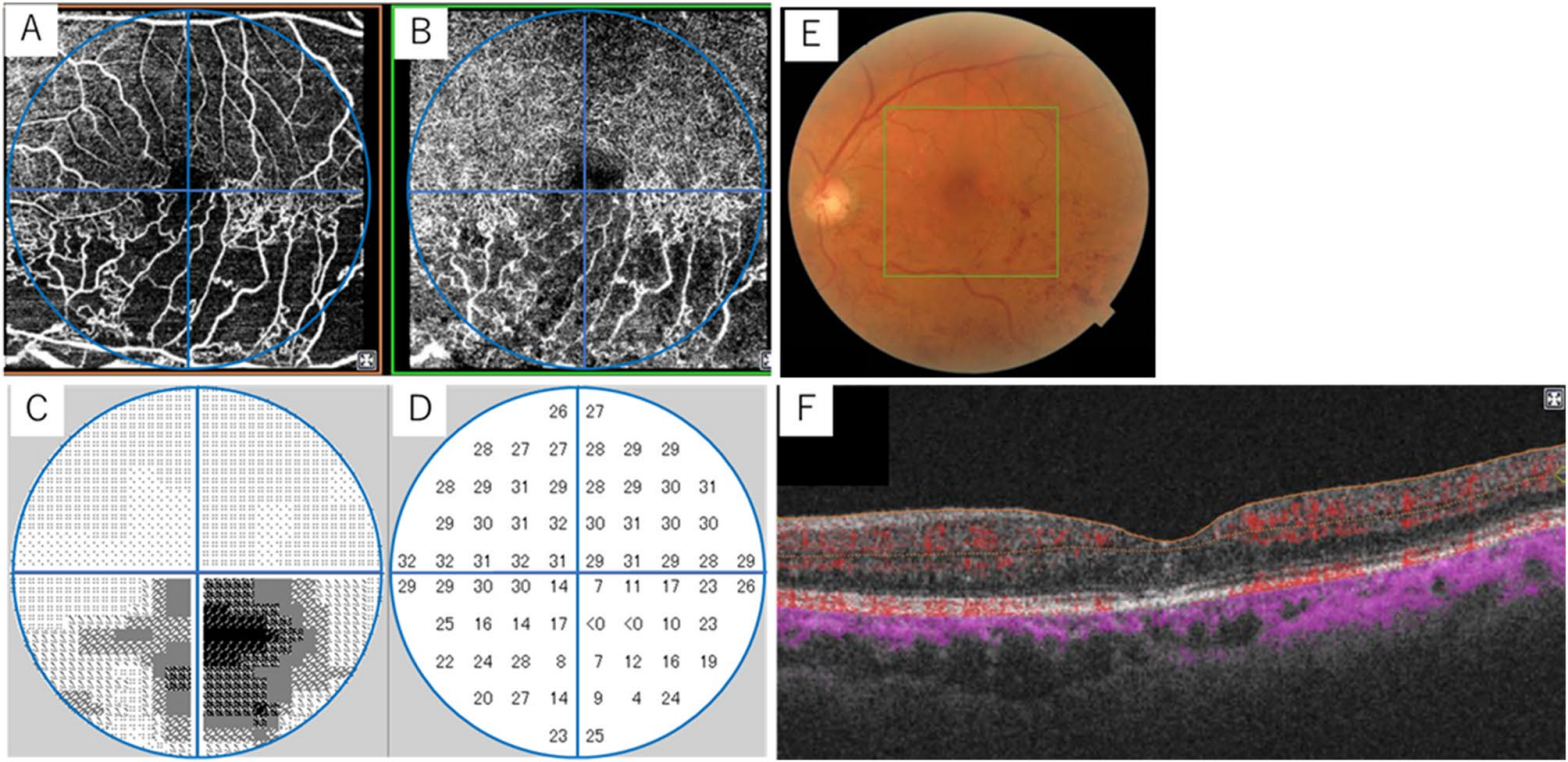
The representative case is the left eye (visual acuity, 20/32) of an 80-year-old male patient 12 months following treatment with four injections of intravitreal aflibercept for supero-temporal major branch retinal vein occlusion. Upside-down images of 6 × 6 mm optical coherence tomography angiography in the superficial capillary plexus (**A**), deep capillary plexus (**B**), and colour fundus photograph (**E**). (**C**) represents the grey scale of the Humphrey visual field analyser 10-2 programme, and (**D**)represents the measured threshold. F shows the swept-source optical coherence tomography image with complete oedema absorption. In OCT angiography, these areas are represented as m-NPA, but the visual field sensitivity does not point to uniformly absolute scotomas, indicating varying sensitivity. *m-NPA* macular non-perfused areas, *OCT* optical coherence tomography.

The capillary drop-out area judged as the m-NPA on OCT angiography and the CVFS of the corresponding region as measured by HFA 10-2 showed a strong correlation in this study. From these results, it is possible to estimate the extent of retinal ischaemia from declines in the visual field sensitivity area, and it can be concluded that the non-invasive evaluation of macular ischaemic area using HFA10-2 is possible. Additionally, all the m-NPAs measured in this study did not exhibit absolute scotoma, and some areas remained in the reduced sensitivity area (Fig. 3). In a previous study using MAIA, Manabe et al. reported that the retinal sensitivity of parafoveal NPA in BRVO was significantly lower in SCP than in DCP^26^. From this, we assume that, although the retinal sensitivity will not be lost, it will decrease in cases in which only deep capillary obstruction is performed. In contrast, the regions with complete superficial and deep capillary obstruction present absolute scotoma.

**Figure 4:**
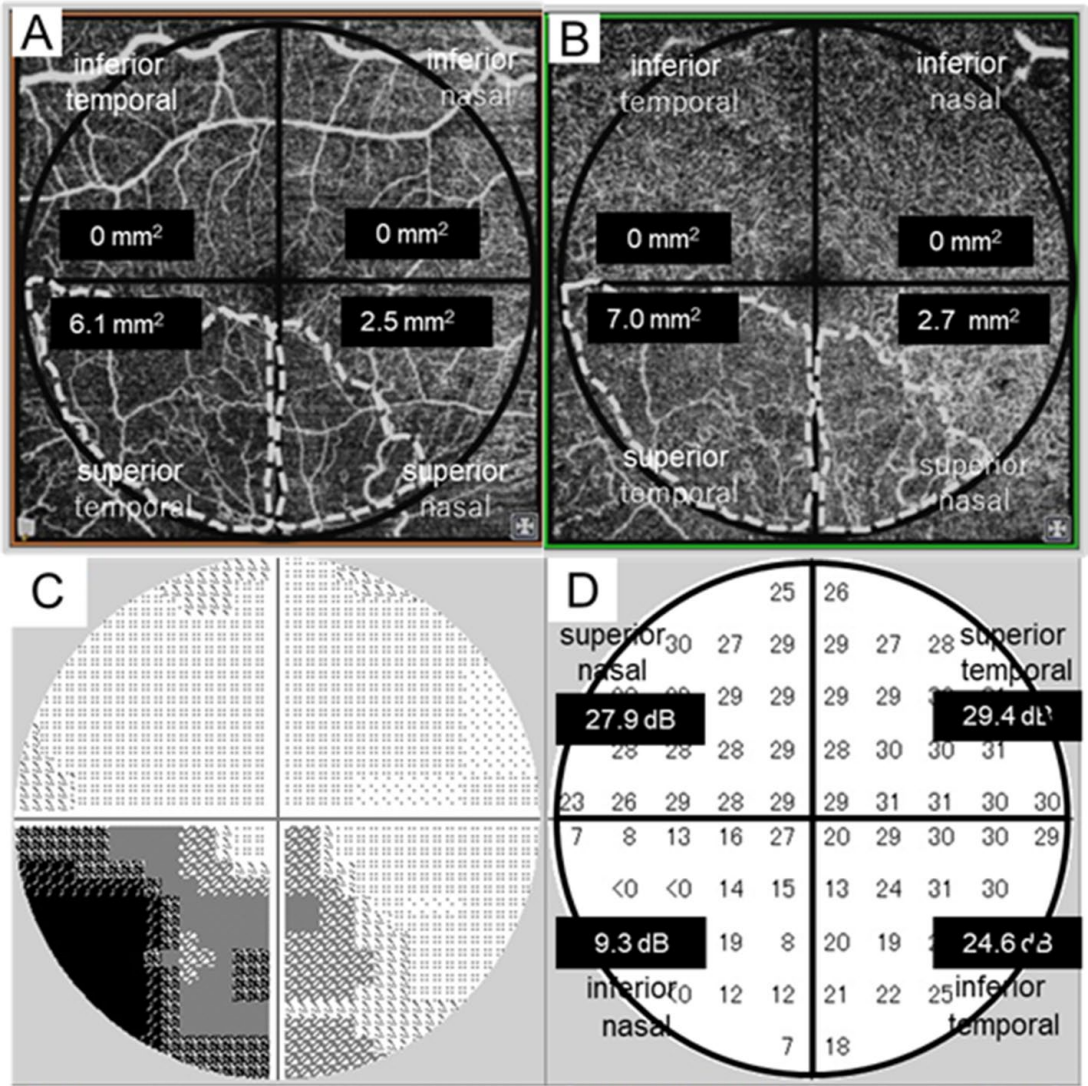
Upside-down images of 6 × 6 mm optical coherence tomography angiography in the superficial capillary plexus (**A**) and deep capillary plexus (**B**). The regions surrounded by the white dotted line indicate the area measured for the evaluation of the macular non-perfusion areas divided into the four-quadrant sectors (superior temporal, superior nasal, inferior temporal, inferior nasal area) in the 6-mm circle except for the foveal avascular zone. (**C**) represents the grey scale of the Humphrey visual field analyser 10-2 programme, and (**D**) represents the measured threshold. Measurement thresholds for all 68 points are organised into the four regions (17 points each: superior temporal, superior nasal, inferior temporal, inferior nasal field), and each mean visual field sensitivity is shown in (**D**). The case is the right eye (visual acuity, 20/25) of a 73-year-old female patient, 12 months following treatment with five injections of intravitreal aflibercept for supero-temporal major branch retinal vein occlusion.

Both the SCP-NPA and DCP-NPA showed a high correlation with the CFVS, and no significant difference was observed between both layers in our results. This is probably because SCP-NPA and DCP-NPA overlapped in most cases and appeared to be connected. In addition, as we evaluated the NPA by region, we estimated that it was difficult to reflect the extent of drop-out of individual capillary vessels. In a study that compared the reduction rate of the blood vessel density between the SCP and DCP using swept-source OCT angiography (3 × 3-mm scans), the degree of ischaemic eye damage was 1.77–1.84 times higher in the DCP than in the SCP with BRVO^27^. Similarly, in our results, no statistically significant difference was observed. Still, DCP-NPA had a larger area in all four regions than SCP-NPA (Table 2), and it seems that DCP was more affected by blood flow in BRVO. Conventionally, in the evaluation of NPA in FA, only SCP is observed, and DCP is not evaluated. Therefore, further research is needed to assess the state of capillary blood flow by layer and the corresponding retinal function using OCT angiography. As a precaution at the time of measurement, if CME or SRD exist, it is difficult to visualise blood vessels due to segmentation errors or blocks^28^. Likewise, in the HFA, if SRD exists, the sensitivity significantly decreases, and the evaluation of the presence or absence of retinal ischaemia becomes difficult. The timing of both examinations is based on data measured after the complete resolution of CME and SRD after intravitreal ranibizumab injection (IVR) or intravitreal aflibercept injection (IVA) treatment, therefore, the results are considered highly credible. However, it was difficult to judge the presence or absence of NPAs in cases without macular oedema resolution, or in which the lesion was found in the anterior ocular segment, which showed intermediate translucency, or in which the image was obtained using OCT angiography. Particularly, the presence of CME, SRD, and residual bleeding led to a lack of clarity of the DCP image, resulting in its exclusion. For HFA10-2 examination, each case met the test reliability criteria, and test reproducibility was confirmed in BRVO patients. Particularly, the grey scale was useful for visually representing NPA (Figs. 3C, 4C). In addition to allowing for the investigation of the presence or absence of macular region ischaemia, as in this study, HFA10-2 is useful for the evaluation of macular function. As shown in Fig. 3, the visual field sensitivity was not uniformly decayed in the areas that were judged as being m-NPAs in the SCP or DCP. However, a difference was observed in the degree of sensitivity reduction, and it was confirmed that the visual function remained. In cases in which grid photocoagulation is performed on the macula, we recommend that HFA examination be conducted in advance, for the establishment of retina sensitivity, and, thereby, for the prevention of coagulation in the remaining function site.

Currently, microperimetry can only be used in limited facilities, while HFA is widely used. Therefore, we believe that HFA is more versatile and practical for the non-invasive evaluation of macular ischaemia. It may be more useful especially in facilities where FA cannot be performed or in which OCT angiography is not installed.

One of the major limitations of this study is the small sample size. The measured NPAs in both SCP and DCP by three examiners demonstrated excellent reliability with mean intraclass correlation coefficient (ICC) of 0.976 and 0.971, respectively. However, if the software in swept-source OCT that automatically measures the NPAs is introduced, the objectivity of the measured data will further increase. Besides, although the loss of capillaries was evaluated by area in this study, it seems that more evaluation that is accurate may be possible using the density of blood vessels. The software attached to the current swept-source OCT cannot measure the blood vessel density in the 6 × 6 mm range. With future technological advances, it will be possible to confirm the correlation between vascular density and retinal sensitivity in SCP and DCP in a large number of cases.

## Conclusion

OCT angiography was useful in the evaluation of m-NPAs in BRVO. The visual field sensitivity reduction region observed on HFA 10-2 reflects the non-perfusion region with a strong correlation. Although both examinations are almost equally localised, macular ischaemia can be estimated non-invasively. The use of these examinations in combination allows for the evaluation of the vascular perfusion state and macular function of the corresponding site.

## Methods

This retrospective, non-randomised study included 30 eyes of 30 consecutive patients with treatment-naïve BRVO-CME. All cases had received treatment with IVR (0.5 mg) or IVA (2 mg) at the Niigata University Hospital between May 2014 and October 2018. Eyes were excluded if the patient had any other ocular pathology that could affect the visual field sensitivity and OCT image acquisition (e.g. Emery-Little classification grade III cataract or greater, glaucoma, optic nerve disease, or DR). All patients underwent comprehensive ophthalmologic evaluations, including the measurement of the BCVA, slit-lamp biomicroscopy, colour fundus photography, swept-source OCT, swept-source OCT angiography, and HFA 10-2, all on the same day. The time of evaluation in this study was defined as the time when BRVO macular oedema and haemorrhage was entirely resolved by treatment. This study received approval from the Research Ethics Committee of Niigata University Hospital prior to initiation, and followed the tenets of the Declaration of Helsinki. Informed consent was obtained from all subjects in this study. Based on fundus examinations, the patients were classified as ‘major BRVO’ and ‘macular BRVO’. Retinal microstructures were obtained using swept-source OCT (DRI OCT-1, Topcon, Japan) by way of 12-mm radial scan protocols, and CRT values were recorded for analysis from the retinal thickness maps.

### Swept-source OCT angiography measurement

Swept-source OCT angiography was performed at CME and SRD resolution after IVR or IVA in all cases. For the evaluation of the m-NPA, data were taken from the first instance of oedema elimination, and a clear OCT angiography image was obtained and adopted. OCT angiography was used to photograph the macula at a 6 × 6-mm range, and the SCP slab was taken from the inner limiting membrane or the vitreoretinal interface and the inner plexiform layer for the incorporation of the ganglion cell layer. The DCP slab was taken from the inner plexiform layer and the outer plexiform layer for the incorporation of the inner nuclear layer. Three examiners measured the size of the capillary drop-out area as the m-NPA of each of the four-quadrant sectors (superior temporal, superior nasal, inferior temporal, inferior nasal area) in a 6-mm circle except for the foveal avascular zone. The m-NPA was manually marked in the SCP and DCP on en face OCT angiography images and measured in square millimetres using measurement software, (Figs. 4A, B). The measurement of the m-NPAs for each case was performed independently by three fully trained examiners, who did not know any other information regarding the study eyes. The inter-examiner reliability was very good, with ICC of 0.976 in SCP and 0.971 in DCP.

### Central visual field sensitivity measurement

To assess the CVFS, HFA 10-2 (Humphrey visual field Analyser II, 10-2 Swedish Interactive Thresholding Algorithm, Humphrey-Zeiss instrument, Dublin, CA, USA) was used to measure the thresholds at all 68 points. HFA 10-2 was performed on the same day with OCT angiography. For the regional analysis corresponding to the fundus, the mean value of all 68 points was defined as the CVFS for the entire macular field. The measurement thresholds for all 68 points were organised into the four regions (17 points each; superior temporal, superior nasal, inferior temporal, inferior nasal field), and the mean value for each region was used (Fig. 4C, D). Only eyes with reliable HFA 10-2 values, defined as fixation losses of 33% or lower, false positives of 15% or lower, and false negatives of 20% or lower were included.

The HFA 10-2 measurement region and the OCT angiography analysis region (6 mm) were regarded as equivalent; analyses were performed in the range corresponding to each of the four regions of the 6-mm circle.

To evaluate the reproducibility of the HFA10-2 examination, HFA was measured twice (test 1 and 2) on the same day in 11 BRVO patients. The mean CVFS values (34 points each; superior and inferior field) of the BRVO-affected and non-affected macular areas were compared between tests 1 and 2. We confirmed the reproducibility of HFA10-2 by testing the difference in mean values per half-field of the two tests.

### Statistical analysis

We compared the mean values and calculated the standard deviations for each visual acuity value and CRT. BCVA was converted to logMAR values using decimal visual acuity values. The unpaired t test was used to compare the mean m-NPAs between the SCP and DCP, and the affected and non-affected areas. Chi-squared tests were used to test the association between categorical variables. To evaluate the reproducibility of HFA10-2, the Wilcoxon signed rank test was used to compare the mean CVFS between tests 1 and 2 in the affected and non-affected areas. The correlation between the m-NPA and CVFS was analysed using Pearson’s correlation coefficient. Intraclass correlation coefficients (ICCs) were used to assess the reliability between the examiners. All statistical analyses were performed using SPSS software version 21.0 (SPSS Inc, Chicago, IL, USA). P values < 0.05 were considered statistically significant.

## Supplementary Information

Supplementary Information.

## Data Availability

The datasets generated during and/or analysed during the current study are not publicly available.

## References

[1] Glacet-Bernard A (1996). Prognostic factors for retinal vein occlusion. Ophthalmology.

[2] Rogers SL (2010). Natural history of branch retinal vein occlusion: an evidence-based systematic review. Ophthalmology.

[3] Yannuzzi LA (1986). Fluorescein angiography complication survey. Ophthalmology.

[4] Imai A, Toriyama Y, Iesato Y, Hirano T, Murata T (2014). En face swept-source optical coherence tomography detecting thinning of inner retinal layers as an indicator of capillary nonperfusion. Eur. J. Ophthalmol..

[5] Sakimoto S (2015). Analysis of retinal nonperfusion using depth-integrated optical coherence tomography images in eyes with branch retinal vein occlusion. Invest. Ophthalmol. Vis. Sci..

[6] Shin YU, Lee BR, Kim S, Lee WJ (2012). A novel noninvasive detection method for retinal nonperfusion using confocal red-free imaging. Ophthalmology.

[7] Hwang TS (2016). Automated quantification of capillary nonperfusion using optical coherence tomography angiography in diabetic retinopathy. JAMA Ophthalmol..

[8] De Salles MC, Kvanta A, Amrén U, Epstein D (2016). Optical coherence tomography angiography in central retinal vein occlusion: correlation between the foveal avascular zone and visual acuity. Invest. Ophthalmol. Vis. Sci..

[9] Coscas F (2016). Optical coherence tomography angiography in retinal vein occlusion: evaluation of superficial and deep capillary plexa. Am. J. Ophthalmol..

[10] Sellam A (2017). Qualitative and quantitative follow-up using optical coherence tomography angiography of retinal vein occlusion treated with anti-VEGF. Retina..

[11] Kuehlewein L, An L, Durbin MK, Sadda SR (2015). Imaging areas of retinal nonperfusion in ischemic branch retinal vein occlusion with swept-source OCT microangiography. Ophthalmic. Surg. Lasers Imaging Retina..

[12] Kashani AH, Lee SY, Moshfeghi A, Durbin MK, Puliafito CA (2015). Optical coherence tomography angiography of retinal venous occlusion. Retina..

[13] Suzuki N (2016). Microvascular abnormalities on optical coherence tomography angiography in macular edema associated with branch retinal vein occlusion. Am. J. Ophthalmol..

[14] Moussa M (2019). Grading of macular perfusion in retinal vein occlusion using en-face swept-source optical coherence tomography angiography: a retrospective observational case series. BMC Ophthalmol..

[15] Asaoka R (2014). Mapping glaucoma patients’ 30-2 and 10-2 visual fields reveals clusters of test points damaged in the 10-2 grid that are not sampled in the sparse 30–2 grid. PLoS ONE.

[16] Iikawa R (2020). Estimation of the central 10-degree visual field using en-face images obtained by optical coherence tomography. PLoS ONE.

[17] Traynis I (2014). Prevalence and nature of early glaucomatous defects in the central 10° of the visual field. JAMA Ophthalmol..

[18] Park SC (2013). Parafoveal scotoma progression in glaucoma: Humphrey 10-2 versus 24-2 visual field analysis. Ophthalmology.

[19] Ota M (2012). Retinal sensitivity after resolution of the macular edema associated with retinal vein occlusion. Graefe’s Arch. Clin. Exp. Ophthalmol..

[20] Lim HB, Kim MS, Jo YJ, Kim JY (2015). Prediction of retinal ischemia in branch retinal vein occlusion: spectral-domain optical coherence tomography study. Invest. Opthalmol. Vis. Sci..

[21] Terashima H, Okamoto F, Hasebe H, Matsuoka N, Fukuchi T (2018). Vitrectomy for epiretinal membranes: ganglion cell features correlate with visual function outcomes. Ophthalmol. Retina..

[22] Chee CKL, Flanagan DW (1993). Visual field loss with capillary non-perfusion in preproliferative and early proliferative diabetic retinopathy. Br. J. Ophthalmol..

[23] Tomiyasu T (2019). Structural and functional analyses of retinal ischemia in eyes with retinal vein occlusion: Relationship with macular edema or microaneurysm formation. Ophthalmic Res..

[24] Kadomoto S (2017). Evaluation of macular ischemia in eyes with branch retinal vein occlusion. Retina..

[25] Yamaike N (2009). Retinal sensitivity after intravitreal injection of bevacizumab for the treatment of macular edema secondary to retinal vein occlusion. Retina..

[26] Manabe S (2017). Association between parafoveal capillary nonperfusion and macular function in eyes with branch retinal vein occlusion. Retina..

[27] Kim JT, Chun YS, Lee JK, Moon NJ, Yi DY (2020). Comparison of vessel density reduction in the deep and superficial capillary plexuses in branch retinal vein occlusion. Ophthalmologica..

[28] Adhi M (2016). Retinal capillary network and foveal avascular zone in eyes with vein occlusion and fellow eyes analyzed with optical coherence tomography angiography. Invest. Opthalmol. Vis. Sci..

