# Supplementary material for: Optical coherence tomography angiography and Humphrey field analyser for macular capillary non-perfusion evaluation in branch retinal vein occlusion
Source: Sci Rep. 2021 Feb 25;11:4583. doi: 10.1038/s41598-021-84240-7 (PMC7907134; doi:10.1038/s41598-021-84240-7)
Supplement: Supplementary file 1 — Supplementary Information. [file 41598_2021_84240_MOESM1_ESM.docx]

**Optical coherence tomography angiography and Humphrey field analyser for macular capillary non-perfusion evaluation in branch retinal vein occlusion**

**Hiroko Terashima**^1*^**, Fumiki Okamoto**^2^**, Hiruma Hasebe**^1^**, Eriko Ueda**^1^**, Hiromitsu Yoshida**^1^**, Takeo Fukuchi**^1^

**^1^Division of Ophthalmology and Visual Science, Graduate School of Medical and Dental Sciences, Niigata University, Japan**

**^2^Department of Ophthalmology, Faculty of Medicine, University of Tsukuba, Japan**

**Supplementary Table 1.** **Correlation between NPA in OCT angiography and central visual field sensitivity in HFA10-2**

| SCP | superior temporal | superior nasal | inferior temporal | inferior nasal |
| --- | --- | --- | --- | --- |
| p | <.001 | <.001 | <.001 | <.001 |
| r | -0.83 | -0.64 | -0.73 | -0.79 |
| DCP | **superior temporal** | **superior nasal** | **inferior temporal** | **inferior nasal** |
| p | <.001 | <.001 | <.001 | <.001 |
| r | -0.82 | -0.71 | -0.71 | -0.70 |

NPA = non-perfusion areas; OCT = optical coherence tomography; HFA10-2 = Humphrey visual field analyser 10-2 program; SCP = superficial capillary plexus; DCP = deep capillary plexus; r = Pearson’s correlation coefficient.
